# Could a multimodal fusion model integrating CT radiomics and systemic inflammatory markers improve preoperative risk stratification of parotid masses? A retrospective exploratory study

**DOI:** 10.3389/fimmu.2026.1842957

**Published:** 2026-07-01

**Authors:** Feng Zhao, Jiaxin Liu, Jingwen Ling, Zongyu Li, Guoqiang Chen, Gang Chen, Jiping Su

**Affiliations:** 1 Department of Otolaryngology, Head and Neck Surgery, The First Affiliated Hospital of Guangxi Medical University, Nanning, China; 2 The School of Information and Management, Guangxi Medical University, Nanning, China; 3 Department of Pathology, The First Affiliated Hospital of Guangxi Medical University, Nanning, China

**Keywords:** lymphocyte-to-monocyte ratio (LMR), multimodal fusion, parotid masses, radiomics, risk stratification

## Abstract

**Objectives:**

To develop and internally validate a multimodal fusion model integrating clinical features, lymphocyte-to-monocyte ratio (LMR), and venous-phase CT radiomic data for preoperative benign-versus-malignant risk stratification of parotid masses (PMs), and to exploratorily evaluate its potential adjunctive role relative to standard diagnostic modalities including fine-needle aspiration biopsy (FNAB).

**Methods:**

A retrospective analysis of 490 patients with histopathologically confirmed PMs (2013–2022) was performed, with random assignment to training (70%) and internal validation (30%) cohorts. Radiomic features from venous-phase CT were selected via t-tests, mRMR, and LASSO regression, with all feature selection performed strictly within training folds. Eleven machine learning models were trained and optimized by 10-fold cross-validation, and the model with the highest cross-validated AUC was selected as the optimal radiomic signature. The previously established LMR-based clinical–laboratory signature was re-validated, and logistic regression was used to fuse the two signatures into a multimodal model. Model performance was assessed using receiver operating characteristic analysis, Hosmer–Lemeshow test, calibration curves, and decision curve analysis (DCA). As an exploratory secondary analysis, in 135 patients with preoperative FNAB, the model’s risk estimates were compared against retrospectively assigned MSRSGC categories using DeLong’s test, net reclassification improvement (NRI), and integrated discrimination improvement (IDI).

**Results:**

The multimodal model achieved an AUC of 0.931 (95% CI: 0.888–0.973) in the internal validation set, outperforming the clinical–laboratory signature (AUC = 0.761; p < 0.001) and radiomic signature (AUC = 0.919; p = 0.038), with sensitivity of 0.957, specificity of 0.782, PPV of 0.449, and NPV of 0.990. In an exploratory secondary analysis of the FNAB subset, the model achieved an AUC of 0.952 (95% CI: 0.918–0.987), with higher statistical discrimination than retrospectively assigned MSRSGC categories (AUC = 0.712; p < 0.001; categorical NRI = 0.399, IDI = 0.386, both p < 0.001).

**Conclusion:**

The multimodal fusion model demonstrated improved statistical risk discrimination over single-modal approaches in this retrospective cohort. The model is an investigational adjunctive risk-stratification instrument that is not intended to replace FNAB, MRI, or histopathologic diagnosis. These preliminary findings require prospective multicenter validation before clinical consideration.

## 1 Introduction

Accurate preoperative differentiation between benign and malignant parotid masses (PMs) is essential for guiding treatment strategy, including the extent of surgery, the need for neck dissection or adjuvant therapy, and prognostication. However, achieving reliable preoperative discrimination remains challenging, particularly for low-grade malignancies whose imaging and clinical features may closely resemble those of benign parotid masses (BPMs) (1). In selected cases, a definitive diagnosis may remain difficult even after complete surgical excision and thorough histopathologic examination.

The current diagnostic workup for parotid masses integrates clinical examination, imaging studies, fine-needle aspiration biopsy (FNAB), multidisciplinary evaluation, and definitive histopathologic diagnosis (2). FNAB, although a cornerstone of preoperative cytopathologic assessment, has recognized limitations including variable sensitivity (3) and difficulty characterizing low-grade malignancies whose bland cytology may mimic benign aspirates (4). These limitations have motivated interest in complementary noninvasive approaches that may provide additional information for preoperative risk assessment.

Among the noninvasive modalities available for parotid mass evaluation, cross-sectional imaging plays a central role. MRI is often preferred for its superior soft-tissue characterization and assessment of deep lobe involvement, perineural spread, and the tumor–facial nerve relationship. CT offers advantages in accessibility, shorter acquisition time, lower cost, and better detection of calcifications, and remains widely used in many clinical settings. However, conventional CT assessment relies largely on subjective interpretation of morphological features such as tumor margin and location, introducing interobserver variability and leaving quantitative tumor information unextracted (2).

Radiomics offers a potential complement to conventional imaging: it enables high-throughput extraction of quantitative, sub-visual features that capture intratumoral heterogeneity and may provide information beyond visual assessment (5). Several studies have explored CT-based radiomics for the evaluation of parotid tumors (6–14). However, the clinical application of standalone radiomic models is limited by several factors, including potential feature instability across different scanners and imaging protocols, complex feature selection processes, and relatively small sample sizes that may limit generalizability (1, 15).Complementary information from systemic biomarkers may help address some of these limitations, motivating the integration of hematological markers with radiomic features.

Growing evidence suggests that systemic inflammatory status, as reflected by hematological markers such as the lymphocyte-to-monocyte ratio (LMR), may be associated with malignant transformation in parotid neoplasms (16, 17). LMR reflects the balance between two leukocyte populations implicated in tumor-related immune responses and is routinely obtainable from preoperative blood counts. Our prior work developed and validated a clinical–laboratory model combining clinical features with LMR, achieving an AUC of 0.754, and found that reduced LMR was associated with malignant parotid masses (MPMs). However, LMR is a nonspecific systemic marker that can be influenced by concurrent infection, autoimmune conditions, and other inflammatory states, and previous studies have not uniformly found LMR to discriminate between benign and malignant parotid tumors (18). Despite the promise of this approach, the clinical–laboratory model alone demonstrated only moderate discriminatory capacity, indicating that additional complementary data modalities are needed to further improve risk stratification.

Multimodal data fusion combines complementary information from different data types to improve predictive stability when individual modalities alone are insufficient (19). Both clinical–laboratory and standalone radiomic approaches have recognized limitations, and their combined use may offer incremental benefits for risk stratification. Against this background, we enrolled 490 patients with histopathologically confirmed PMs to develop and internally validate a multimodal risk-stratification model, integrating clinical variables, LMR, and CT-derived radiomic features via logistic regression-based late fusion. Our objectives were to: (1) evaluate whether this combined approach improves preoperative benign-versus-malignant risk stratification of PMs compared with single-modal approaches; (2) comprehensively assess model performance via discrimination, calibration, and clinical utility analyses; and (3) as an exploratory secondary aim, examine how model-based risk estimates relate to FNAB-based cytopathologic classification (the Milan System for Reporting Salivary Gland Cytopathology, MSRSGC) in the subset of patients with available preoperative FNAB data.

## 2 Materials and methods

### 2.1 Participants

**Figure 1:**
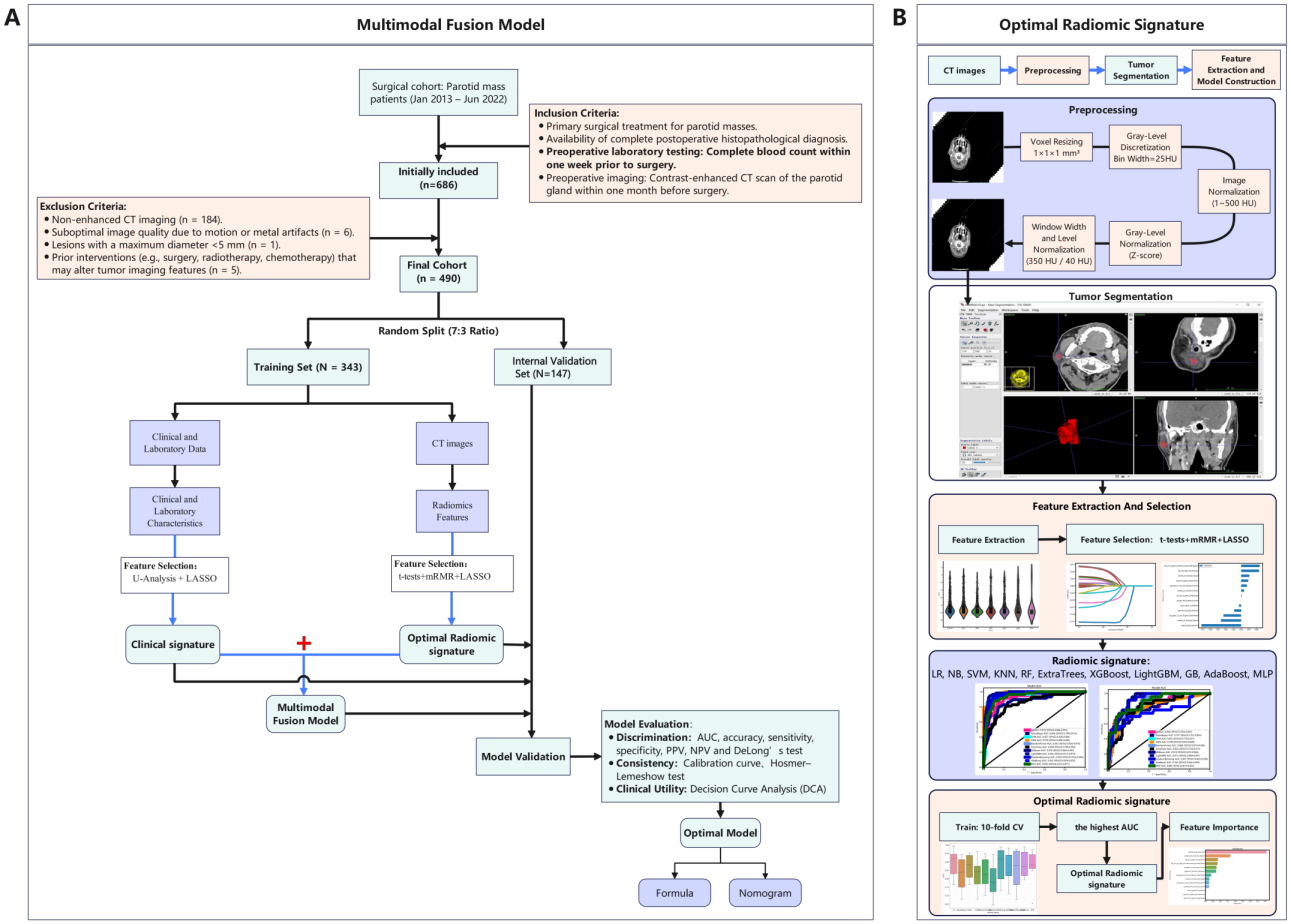
Research design flow chart.

A total of 490 patients who underwent surgical resection for parotid masses and received postoperative pathological confirmation at the First Affiliated Hospital of Guangxi Medical University between January 2013 and June 2022 were included. Tumors were categorized as benign or malignant based on the 2022 WHO classification of salivary gland tumors (20). The patient selection process is summarized in **Figure 1**. The inclusion criteria were: (1) parotid gland lesions confirmed through surgical intervention; (2) high-resolution venous-phase CT imaging performed no more than 30 days before the operation. Cases were excluded if: (1) CT images were compromised by movement or metallic artifacts, resulting in reduced diagnostic quality; (2) lesions with a maximum diameter <5 mm, to minimize volume effect-related bias in radiomic feature extraction (21); (3) prior parotid surgery, radiotherapy, or chemotherapy that could alter tumor imaging characteristics; (4) active infection at the time of blood sampling or imaging, as documented in the medical record; (5) diagnosed autoimmune disease, chronic inflammatory disorder, or hematologic malignancy; (6) current corticosteroid or immunosuppressive therapy; and (7) nonstandard or incomplete imaging protocols. This retrospective study was approved by the Medical Ethics Committee of First Affiliated Hospital of Guangxi Medical University (Approval No. 2025-S0606). Informed consent was waived due to the retrospective nature of the study.

### 2.2 Clinical and laboratory data collection

Clinical and laboratory data were collected following previously established protocols (22), including sex, age, presence of pain or pressure symptoms, peripheral facial nerve palsy, smoking history, and LMR. The temporal relationship between blood sampling, imaging, and surgery was recorded when available. For cases in which FNAB was performed prior to CT imaging, the interval between FNAB and imaging was noted when available.

### 2.3 Image acquisition, preprocessing, and harmonization

Each participant received a contrast-enhanced multidetector CT scan extending from the base of the skull to the region above the clavicle (**Supplementary Table 1**). Venous-phase imaging was performed 60–90 seconds after intravenous administration of a non-ionic iodinated contrast agent (350 mg/mL, 1–2 mL/kg, injected at 3–5 mL/s).

To account for variability in scanning protocols across different CT vendors, scanner generations, slice thicknesses, and acquisition parameters, images were preprocessed according to standardized radiomics guidelines (23, 24), including: (1) selection and storage of venous-phase images showing parotid parenchymal enhancement in DICOM format; (2) voxel resampling to 1 × 1 × 1 mm³ isotropic resolution to reduce slice-thickness variability; (3) intensity discretization using a 25 HU bin width; (4) normalization of signal intensity to 1–500 HU; (5) Z-score standardization of grayscale values; (6) application of a fixed window level (40 HU) and width (350 HU) for consistent visualization; (7) manual delineation of tumor regions of interest on axial CT slices by two senior head and neck specialists (with 9 and 13 years of clinical experience), who had no access to the patients’ clinical or histopathological information. For multifocal lesions, the largest lesion confirmed by pathology was selected. One week later, the surgeon with 9 years of experience repeated the segmentation for all 490 volumes of interest (VOIs, **Supplementary Figure 1**). Necrotic and cystic regions within the tumor were included in the VOI as part of the whole-tumor segmentation approach; dental artifacts were visually assessed, and cases with severe artifacts affecting the tumor region were excluded per criterion (1) (8); extraction of radiomic features from VOIs using PyRadiomics in accordance with the Image Biomarker Standardization Initiative (IBSI) (25), including first-order statistics, 3D shape features, and textural features derived from Gray-Level Co-occurrence Matrix (GLCM), Gray-Level Size Zone Matrix (GLSZM), Gray-Level Run Length Matrix (GLRLM), Gray-Level Dependence Matrix (GLDM), and Neighborhood Gray-Tone Difference Matrix (NGTDM) (9); further normalization of eigenvalues via Z-score transformation; and (10) assessment of inter- and intraobserver reproducibility using the intraclass correlation coefficient (ICC). A two-way random-effects model with single-measure ICC (ICC (1, 2)) was used, and features with ICC > 0.75 for both inter- and intraobserver comparisons were retained for model development (26).

### 2.4 Model development and validation

#### 2.4.1 Dataset partitioning

Following random stratification by malignancy status, the dataset was divided into a training set (70%, n = 343) and an internal validation set (30%, n = 147). All model building, feature selection, and hyperparameter tuning were conducted exclusively on the training set; the internal validation set was reserved solely for unbiased evaluation of final model performance.

#### 2.4.2 Clinical–laboratory signature

Building upon our previously established clinical–laboratory signature (22), formulated as:


$$\text{log}\left(\frac{{\text{P}}_{1}}{{1-P}_{1}}\right)=0.36-0.935*{\text{X}}_{1}+0.972*{\text{X}}_{2}+3.66*{\text{X}}_{3}-0.482*{\text{X}}_{4}$$

where *X_1_* to *X_4_* represent smoking history, presence of pain or pressure symptoms, peripheral facial nerve palsy, and LMR, respectively, and *P_1_* denotes the predicted probability of malignancy. To evaluate its generalizability, the model was applied to the internal validation set without re-fitting, generating estimated malignancy risks for each participant.

#### 2.4.3 Radiomics signature development

To mitigate potential bias from class imbalance, the training set was balanced using the Synthetic Minority Oversampling Technique (SMOTE) (27). All feature selection was performed strictly within the training folds to prevent data leakage. Statistically significant features were first identified using independent-sample t-tests on the training data, and the top 20 were ranked using the minimum redundancy maximum relevance (mRMR) algorithm (28, 29). Finally, non-zero coefficients were retained through 10-fold cross-validated least absolute shrinkage and selection operator (LASSO) regression, with feature selection embedded within each cross-validation fold.

Eleven machine learning models were trained:logistic regression (LR), naive bayes (NB), support vector machine (SVM), K-nearest neighbors (KNN), random forest (RF), extremely randomized trees (ExtraTrees), eXtreme Gradient Boosting (XGBoost), Light Gradient Boosting Machine (LightGBM), gradient boosting (GB), adaptive boosting (AdaBoost), and multilayer perceptron (MLP).These models were trained using the selected radiomics features. Hyperparameters were optimized via 10-fold cross-validation on the training set, and the model with the highest cross-validated AUC was selected as the optimal radiomics model (Radiomics Signature). Feature importance rankings were analyzed to improve model interpretability and assess clinical relevance (30).

#### 2.4.4 Multimodal fusion model construction

A late-stage fusion strategy was employed to integrate clinical and radiomic data (31). Specifically, the predicted probabilities from the clinical–laboratory signature (*P*_1_) and radiomics signature (*P*_2_) were combined as two covariates within a logistic regression framework, which was iteratively optimized on the training set. The model parameters included L2 regularization, a convergence threshold of 1e-06, a maximum of 100 iterations, and a regularization strength (C) of 10.0. The resulting multimodal fusion model was visualized as a nomogram to facilitate clinical interpretation.

#### 2.4.5 Model validation and performance comparison

Model performance was evaluated and compared on the internal validation set using the area under the receiver operating characteristic curve (AUC), accuracy, sensitivity, specificity, positive predictive value (PPV), and negative predictive value (NPV). Bootstrap resampling was used to compute 95% confidence intervals for AUC values, and DeLong’s test was applied to assess statistically significant differences between AUCs. Discriminative performance was categorized as low (0.5–0.7), moderate (0.7–0.9), or high (≥0.9) based on AUC thresholds. Model calibration was examined via the Hosmer–Lemeshow goodness-of-fit statistic and visualized with calibration plots. Decision curve analysis (DCA) was carried out to assess the net clinical benefit across a range of clinically relevant threshold probabilities.

### 2.5 Exploratory comparison with FNAB-based cytopathology (prespecified secondary analysis)

Among the 490 patients, 135 (27.6%) had undergone preoperative FNAB with available reports. FNAB results were retrospectively classified according to the Milan System for Reporting Salivary Gland Cytopathology (MSRSGC) (32), producing the following distribution: non-diagnostic (n = 5), benign (n = 99), atypia of undetermined significance (n = 5), suspicious for malignancy (n = 7), and malignant (n = 19).

For binary classification, MSRSGC categories of “suspicious for malignancy” and “malignant” were considered test-positive. The multimodal fusion model was applied to this subset without re-training, and its discriminative performance was compared against MSRSGC classification using ROC analysis and DeLong’s test. Incremental discriminative performance was assessed using categorical NRI, continuous NRI, and IDI. Risk reclassification was evaluated using the MSRSGC Youden-optimal cutoff (0.39) as the stratification boundary.

### 2.6 Statistical analysis

For continuous variables, descriptive statistics were reported either as mean ± standard deviation or median with interquartile range (IQR), based on the data distribution. Comparisons between groups were conducted using independent t-tests for normally distributed data or Mann–Whitney U-tests for non-parametric distributions. Categorical data were expressed in terms of frequency counts and proportions, and group differences were evaluated through chi-square analysis or Fisher’s exact test when expected cell counts were low. The statistical analyses and model development procedures were performed with R version 4.2.3 and Python 3.6, utilizing key libraries including pROC, RMS, scikit-learn, NumPy, and SciPy. A significance level of α = 0.05 was adopted for hypothesis testing.

## 3 Results

### 3.1 Clinical and laboratory characteristics

This study included 490 patients (male: 58.37%, female: 41.63%, median age: 49 years) with PMs, of whom 409 (83.5%) had BPMs (mainly pleomorphic adenomas and Warthin’s tumors), and 81 (16.5%) had MPMs, predominantly mucoepidermoid carcinoma (n = 22), with other subtypes including adenoid cystic carcinoma (n = 9), acinic cell carcinoma (n = 8), and several additional subtypes each represented by fewer than five cases (**Supplementary Table 2**). The benign-to-malignant ratio was approximately 5:1. No significant differences in baseline characteristics (age, sex, smoking, facial paralysis, pain, LMR) were observed between the training and internal validation testing sets (p > 0.05). However, in the training set, malignant cases showed higher prevalence of pain, facial paralysis, and lower LMR (p < 0.05; **Table 1**).

**Table 1:** Comparison of clinical parameters between training and internal validation sets for BPM and MPM patients.

Characteristic	Total dataset	Training set				Internal validation set				*P3*
		All	BPM	MPM	*P_1_*	All	BPM	MPM	*P_2_*	
Participants, n (%)	490	343	285(83.09)	58(16.91)		147	124(84.35)	23(15.65)		0.73
Sex, n (%)					0.803				0.332	0.401
Male	286(58.37)	196(57.14)	162(56.84)	34(58.62)		90(61.22)	78(62.90)	12(52.17)		
Female	204(41.63)	147(42.86)	123(43.16)	24(41.38)		57(38.78)	46(37.10)	11(47.83)		
Peripheral facial paralysis,n (%)					<0.001				0.666	0.163
NO	480(97.96)	334(97.38)	285(100.00)	49(84.48)		146(99.32)	123(99.19)	23(100.00)		
YES	10(2.04)	9(2.62)	0(0.00)	9(15.52)		1(0.68)	1(0.81)	0(0.00)		
Pain/tenderness, n (%)					<0.001				<0.001	0.959
NO	404(82.45)	283(82.51)	244(85.61)	39(67.24)		121(82.31)	109(87.90)	12(52.17)		
YES	86(17.55)	60(17.49)	41(14.39)	19(32.76)		26(17.69)	15(12.10)	11(47.83)		
Smoking status, n (%)					0.773				0.174	0.355
NO	315(64.29)	225(65.60)	186(65.26)	39(67.24)		90(61.22)	73(58.87)	17(73.91)		
YES	175(35.71)	118(34.40)	99(34.74)	19(32.76)		57(38.78)	51(41.13)	6(26.09)		
Age, year	49.0(34.0,59.0)	49.0(34.0,59.0)	49.0(34.0,60.0)	46.0(38.0,57.0)	0.84	51.0(34.0,58.0)	52.0(36.0,59.0)	43.0(37.0,53.0)	0.255	0.934
LMR	3.93(3.08,4.90)	3.93(3.04,4.91)	4.02(3.18,5.09)	3.26(2.54,4.25)	<0.001	4.03(3.19,4.85)	4.17(3.51,4.92)	3.52(2.65,4.00)	0.002	0.474

*P_1_* compares BPM vs. MPM in the training set; *P_2_* shows the same for the internal validation set; *P_3_* evaluates cohort differences between training and internal validation; LMR lymphocyte-to-monocyte ratio.

### 3.2 Extraction and selection of radiomics features

**Figure 2:**
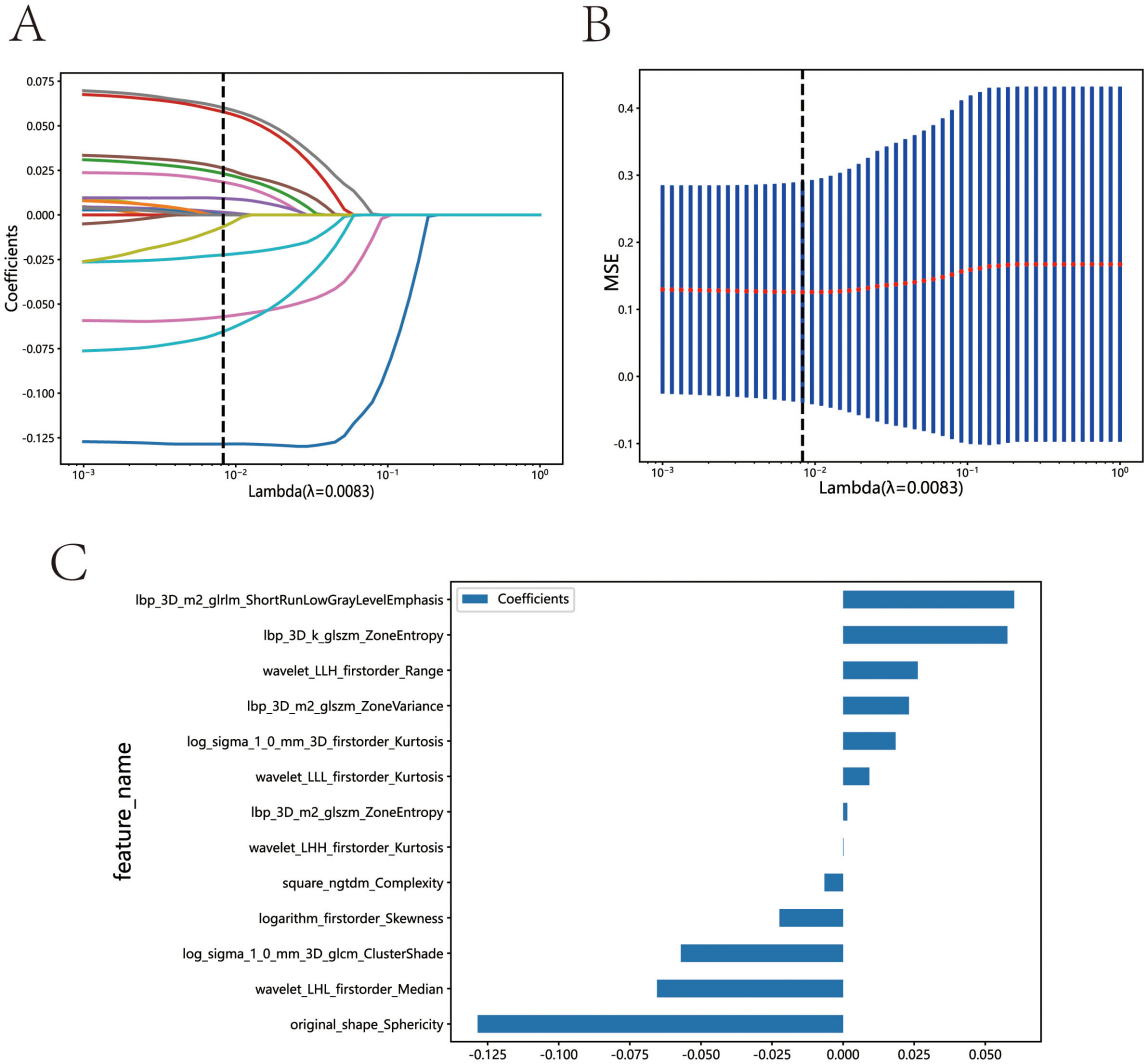
LASSO regression for radiomic feature selection. **(A)** 10-fold cross-validation coefficient map; **(B)** Mean squared error (MSE) with optimal λ (1-SE criterion); **(C)** Final coefficients of the 13 selected features with non-zero values.

A total of 1834 radiomic features were extracted using PyRadiomics. ICC analysis retained 1509 stable features (82.3%; interobserver ICC range: 0.76–0.98; intraobserver ICC range: 0.78–0.99). Independent-sample t-tests reduced this to 879 significant features (**Supplementary Figure 2**). Using mRMR and LASSO regression, 13 predictive features were selected for model construction (**Figure 2**).

### 3.3 Development and evaluation of the optimal radiomics signature

**Figure 3:**
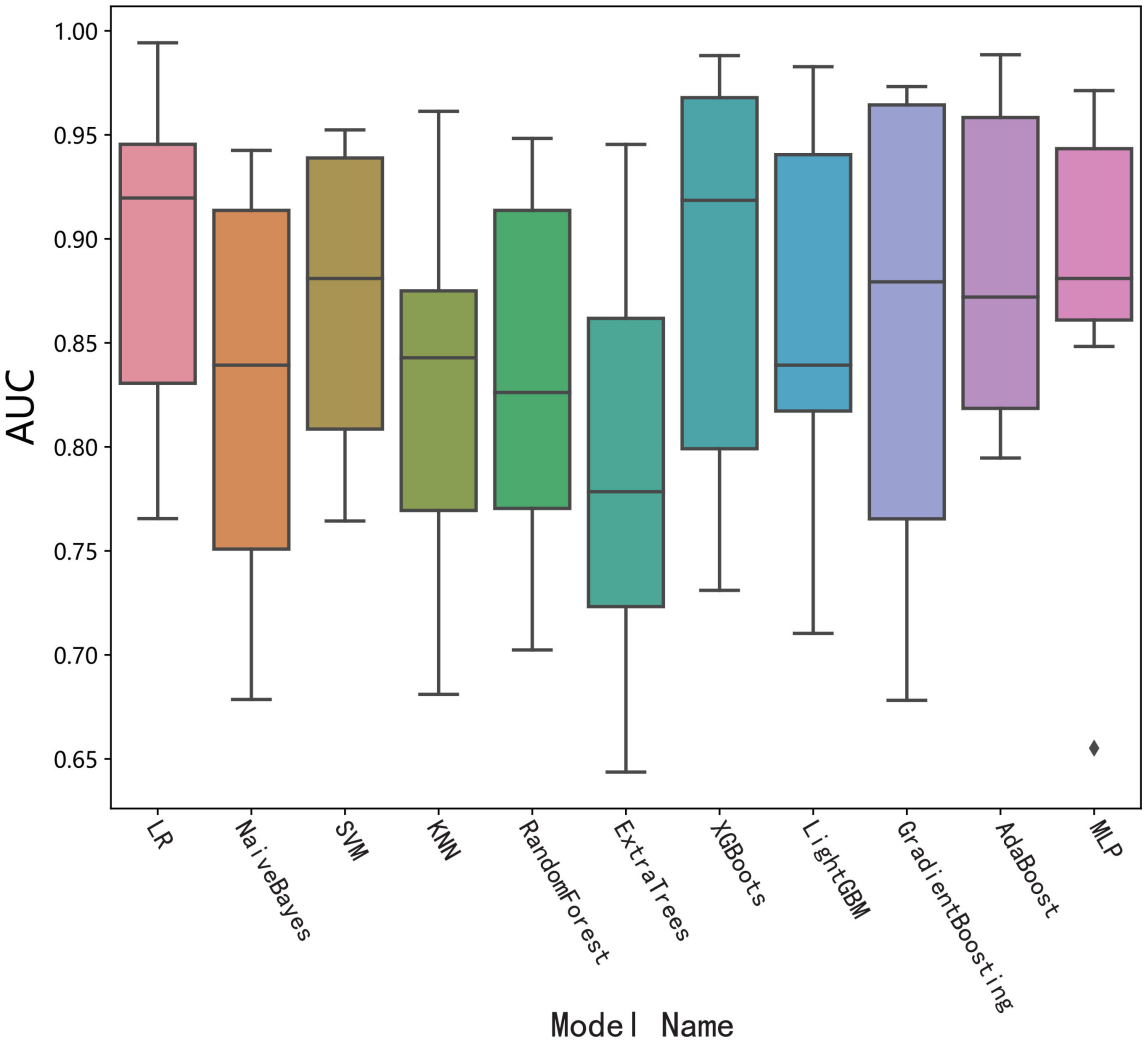
AUC results of 10 fold cross-validation of 11 machine learning models in the training set.

**Figure 4:**
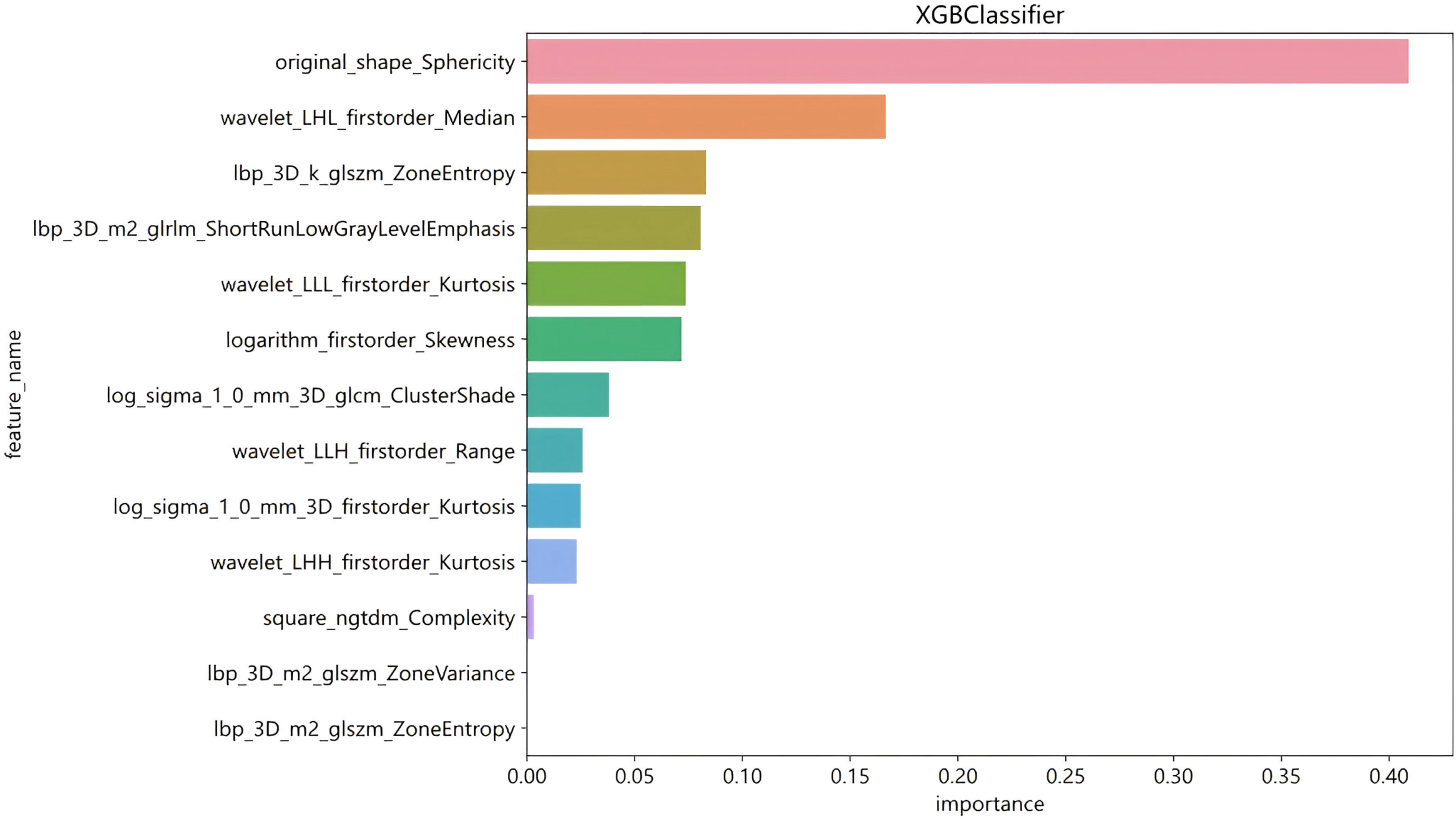
Feature importance ranking for the XGBoost radiomic model.

Using the 13 selected radiomics features, 11 machine learning models were trained on the training set (**Supplementary Table 3**), with hyperparameters optimized via 10-fold cross-validation to minimize overfitting. The XGBoost model achieved the highest cross-validation performance (mean AUC = 0.978) and was selected as the optimal radiomics model (Radiomics Signature, **Figure 3**). Feature importance analysis identified original_shape_Sphericity (0.41), wavelet_LHL_firstorder_Median (0.17), and lbp_3D_k_glszm_ZoneEntropy (0.08) as the top three contributors (**Figure 4**).

### 3.4 Development of the multimodal fusion model

**Figure 5:**
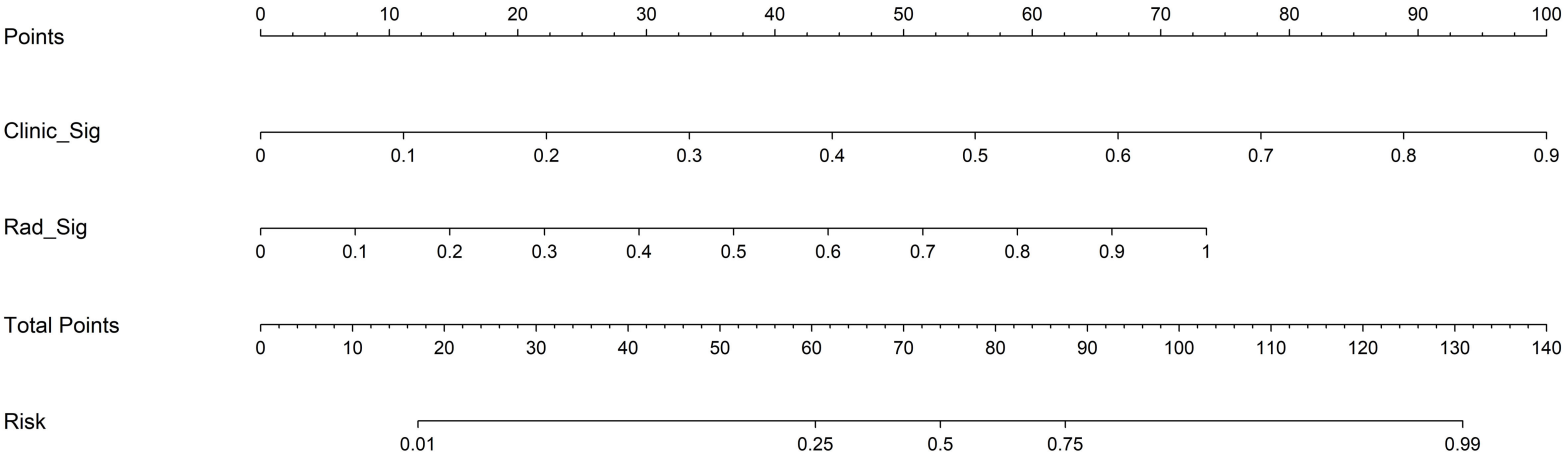
Nomogram of the multimodal fusion model.

The fusion model combined predicted probabilities from the clinical–laboratory signature (*P*_1_) and radiomics signature (*P*_2_) via logistic regression:


$$\text{log}\left(\frac{{\text{P}}_{3}}{{1-P}_{3}}\right)=-7.39+8.66*{\text{P}}_{1}+ 8.21*{\text{P}}_{2}$$

where *P*_3_ represents the final integrated prediction output (**Figure 5**).

### 3.5 Comparison of model performance

#### 3.5.1 Distinction assessment

**Figure 6:**
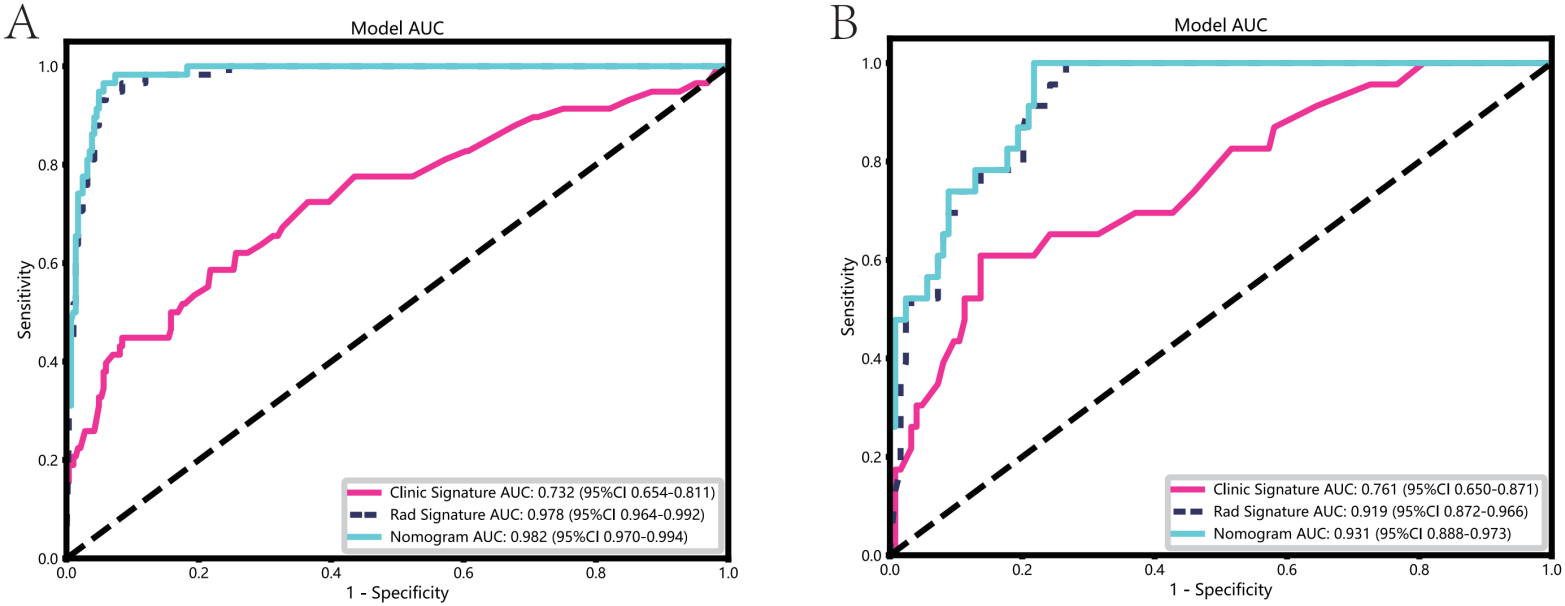
ROC analysis of the clinical, radiomics, and fusion models. **(A)** Training set; **(B)** Internal validation set.

The clinical–laboratory signature yielded an AUC of 0.732 (95% CI: 0.654–0.811) in the training set and 0.761 (95% CI: 0.650–0.871) in the internal validation set. The radiomics signature demonstrated stronger performance with an AUC of 0.919 (95% CI: 0.872–0.966). The multimodal fusion model enhanced discriminatory performance, achieving an AUC of 0.931 (95% CI: 0.888–0.973). It preserved the high sensitivity (0.957) and NPV (0.990) of the radiomics signature, while showing improved specificity (0.782 vs. 0.734) and PPV (0.449 vs. 0.400) (**Figure 6**; **Table 2**). DeLong tests confirmed that the radiomics signature outperformed the clinical signature (p < 0.05), and the fusion model significantly outperformed both in the internal validation set (p < 0.05; **Table 3**).

**Table 2:** ROC analysis of clinical, radiomics, and multimodal models.

Data sets	Models	AUC	95% CI	Accuracy	Sensitivity	Specificity	PPV	NPV
Training set	Clinic Signature	0.732	0.654 - 0.811	0.746	0.552	0.786	0.344	0.896
	Rad Signature	0.978	0.964 - 0.992	0.939	0.931	0.94	0.761	0.985
	Nomogram	0.982	0.970 - 0.994	0.945	0.948	0.944	0.775	0.989
Internal validation set	Clinic Signature	0.761	0.650 - 0.871	0.81	0.522	0.863	0.414	0.907
	Rad Signature	0.919	0.872 - 0.966	0.769	0.957	0.734	0.4	0.989
	Nomogram	0.931	0.888 - 0.973	0.81	0.957	0.782	0.449	0.99

CI indicates confidence interval, PPV indicates positive predictive value; NPV indicates negative predictive value. The term “Nomogram” specifically refers to Multimodal Models.

**Table 3:** AUC comparison of models (DeLong's test).

Model comparison	Training set		Internal validation set	
	Z-value	P-value	Z-value	P-value
Nomogram VS. Clinic Signature	6.221	<0.001	2.921	0.004
Nomogram VS. Rad Signature	1.891	0.059	2.073	0.038
Rad Signature VS. Clinic Signature	5.608	<0.001	2.363	0.018

The table presents the results of pairwise comparisons of AUC values using DeLong's test for both training and internal validation sets.

#### 3.5.2 Calibration assessment

**Figure 7:**
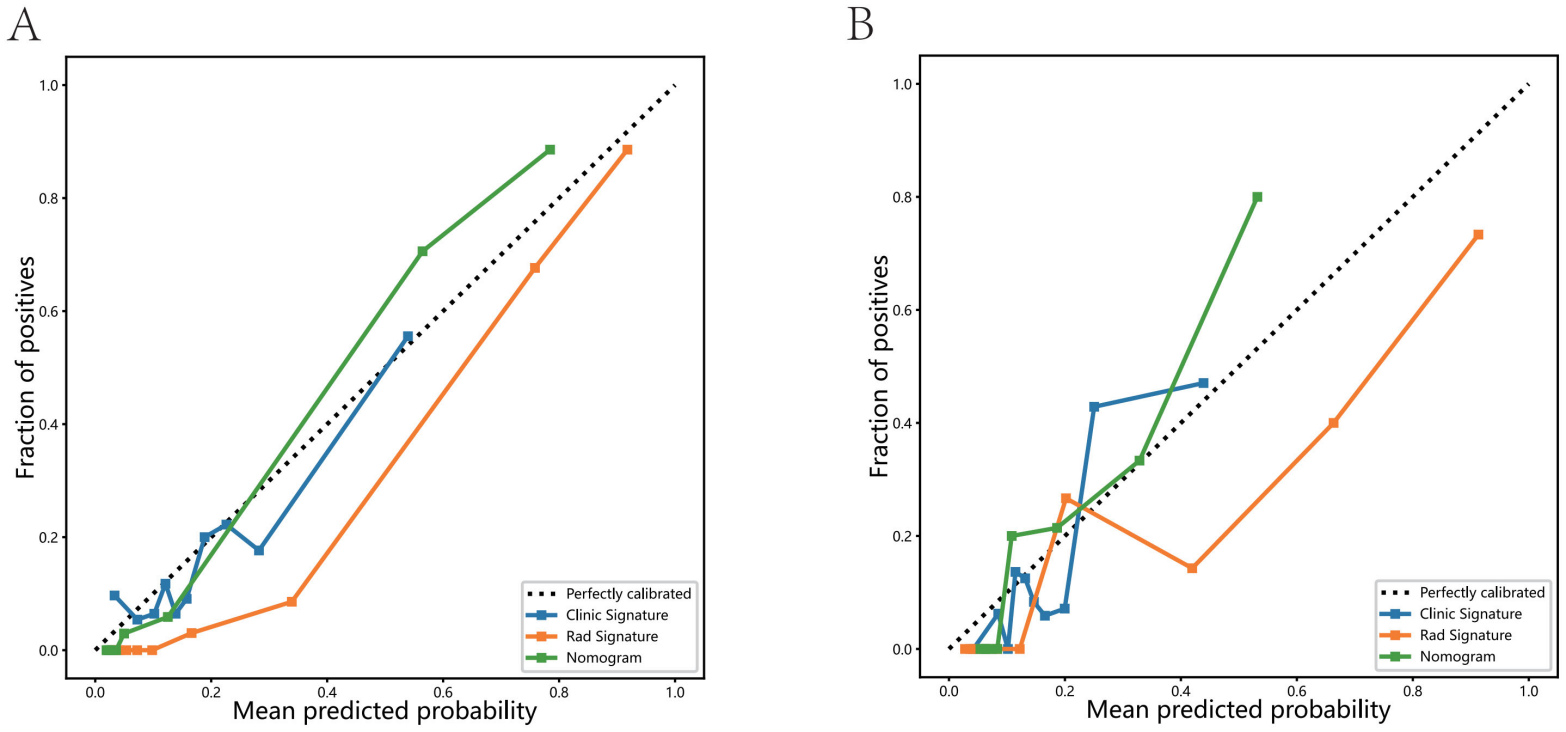
Model calibration curves in the training and internal validation sets. **(A)** Training set; **(B)** Internal validation set.

Hosmer–Lemeshow tests indicated good calibration for the clinical–laboratory signature and fusion model across both datasets, whereas the radiomics signature showed poor calibration (**Table 4**). Calibration curves showed deviations from the ideal diagonal line in the radiomics signature within medium-to-low probability ranges, suggesting suboptimal reliability in low-probability predictions but better accuracy at higher probabilities (**Figure 7**).

**Table 4:** Calibration performance comparison among models (Hosmer-Lemeshow test).

Datasets	Clinic signature	Rad signature	Nomogram
Training set	0.398	<0.001	0.147
Internal validation set	0.837	0.006	0.151

Hosmer–Lemeshow test results for calibration performance of the clinical, radiomics, and nomogram models; lower P-values indicate poorer fit.

#### 3.5.3 Clinical net benefit assessment

**Figure 8:**
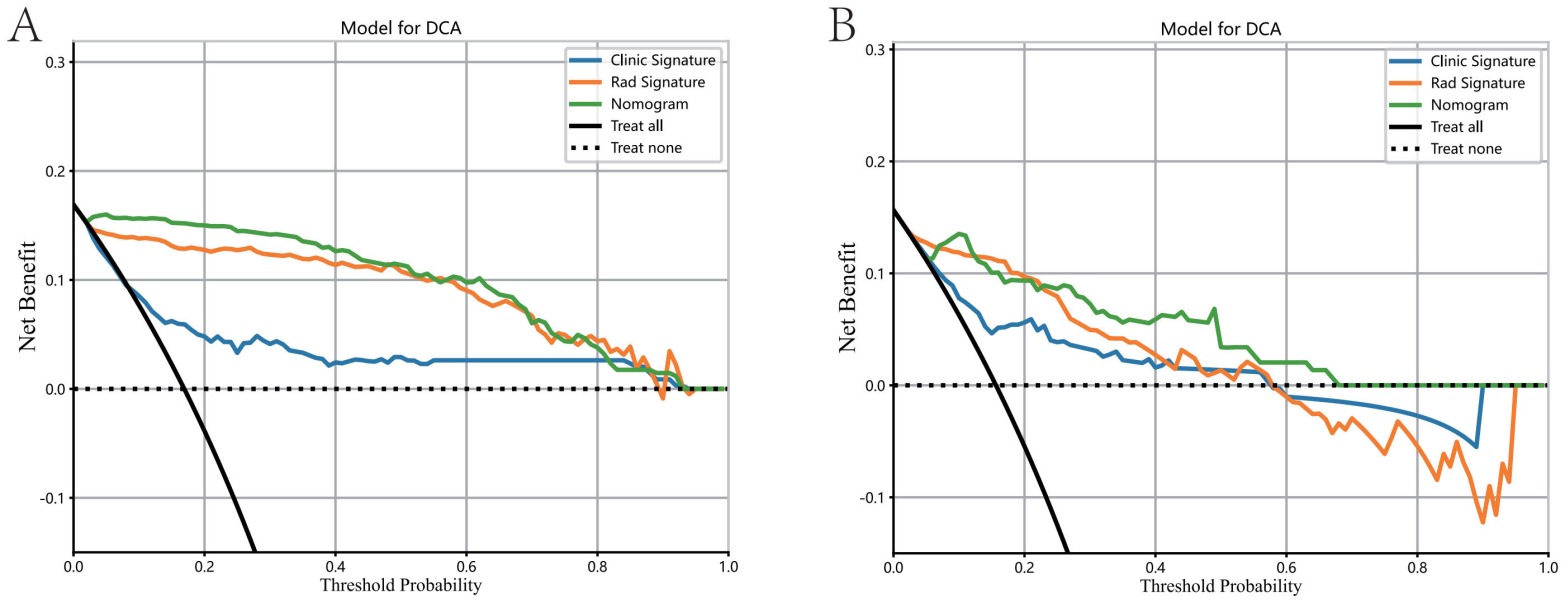
Decision curve analysis (DCA) for the clinical-laboratory signature, radiomics signature, and multimodal fusion model. **(A)** Training set; **(B)** Internal validation set.

According to DCA, the fusion model offered a higher net clinical benefit across applicable threshold probabilities compared to both the clinical–laboratory and radiomics signatures, especially within the range of 0.03–0.93 for the training set and 0.06–0.68 for the internal validation set (**Figure 8**).

### 3.6 Exploratory comparison of the multimodal model with FNAB-based cytopathology

**Figure 9:**
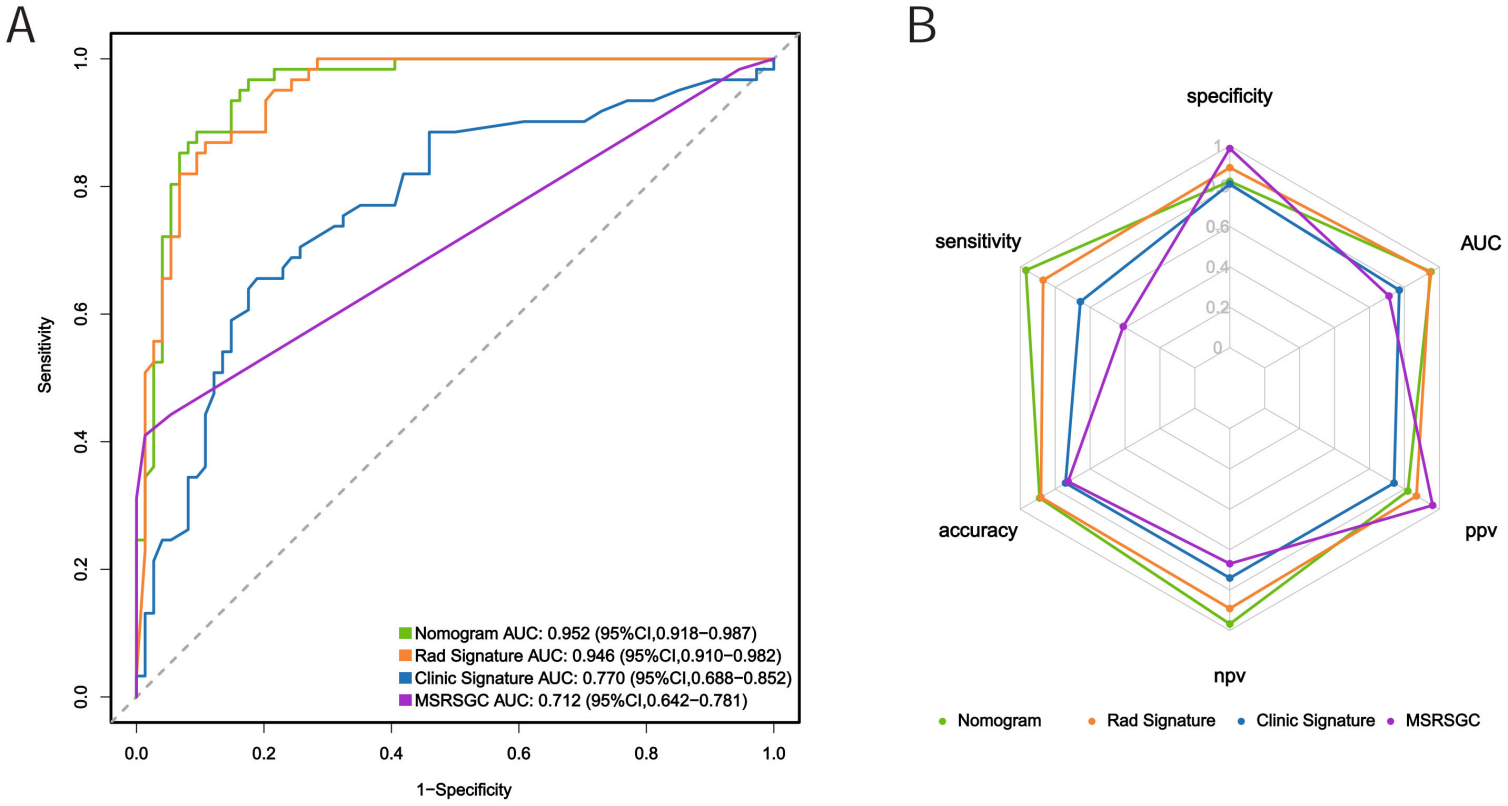
Exploratory comparison of the multimodal model with MSRSGC-based cytopathology in the FNAB subset. **(A)** ROC curves; **(B)** Radar chart of diagnostic performance.

Among the 135 patients with available preoperative FNAB, 74 (54.8%) had benign lesions and 61 (45.2%) had malignant lesions (**Supplementary Table 4**), reflecting the preferential use of FNAB in clinically suspicious cases. According to the MSRSGC classification, the binary discrimination of benign versus malignant parotid masses achieved an AUC of 0.712 (95% CI: 0.642–0.781). When applied to the same subset, the multimodal fusion model achieved an AUC of 0.952 (95% CI: 0.918–0.987; DeLong test: Z = 6.584, p < 0.001; **Figure 9**). At the MSRSGC threshold of “suspicious for malignancy” and above, sensitivity, specificity, accuracy, NPV, and PPV were 0.410, 0.986, 0.726, 0.670, and 0.962, respectively. The multimodal model, at its Youden-optimal cutoff derived from the training set (0.112) and applied without modification to the FNAB subset, yielded corresponding values of 0.967, 0.824, 0.889, 0.968, and 0.819 (**Table 5**).

**Table 5:** Exploratory comparison of the multimodal model with MSRSGC-based cytopathology in the FNAB subset (n = 135).

A. Diagnostic Performance Metrics				
Metric	Nomogram	Rad signature	Clinic signature	MSRSGC
Threshold	0.112	0.453	0.225	3.500
AUC (95% CI)	0.952 (0.918–0.987)	0.946 (0.910–0.982)	0.770 (0.688–0.852)	0.712 (0.642–0.781)
Accuracy	0.889	0.881	0.741	0.726
Sensitivity	0.967	0.869	0.656	0.410
Specificity	0.824	0.892	0.811	0.986
PPV	0.819	0.869	0.741	0.962
NPV	0.968	0.892	0.741	0.670
B. Risk reclassification (MSRSGC Youden cutoff = 0.39)				
Outcome	MSRSGC category	Model low risk	Model high risk	% Reclassified
Benign (n = 74)	Low Risk	64	6	9%
Benign (n = 74)	High Risk	4	0	100%
Malignant (n = 61)	Low Risk	7	27	79%
Malignant (n = 61)	High Risk	1	26	4%
C. Incremental performance indices				
Index	Value (95% CI)	p-value		
Categorical NRI	0.399 (0.243–0.556)	< 0.001		
Continuous NRI	1.385 (1.140–1.630)	< 0.001		
IDI	0.386 (0.289–0.482)	< 0.001		

AUC, area under the receiver operating characteristic curve; CI, confidence interval; FNAB, fine-needle aspiration biopsy; IDI, integrated discrimination improvement; MSRSGC, Milan System for Reporting Salivary Gland Cytopathology; NRI, net reclassification improvement; NPV, negative predictive value; PPV, positive predictive value.

Risk reclassification analysis (MSRSGC Youden cutoff = 0.39) showed that among malignant cases, 79% (27/34) initially classified as low-risk by MSRSGC were reclassified as high-risk by the multimodal model, while among benign cases, all four high-risk MSRSGC classifications were corrected to low-risk (**Table 5**). Incremental diagnostic performance indices confirmed significant improvement: categorical NRI = 0.399 (95% CI: 0.243–0.556, p < 0.001); continuous NRI = 1.385 (95% CI: 1.140–1.630, p < 0.001); and IDI = 0.386 (95% CI: 0.289–0.482, p < 0.001).

## 4 Discussion

### 4.1 Principal findings and clinical positioning

This study developed a multimodal fusion model combining clinical variables, LMR, and venous-phase CT radiomic features to improve preoperative risk stratification of PMs. The fusion model achieved an AUC of 0.931 in the internal validation set, significantly outperforming both the clinical–laboratory signature (AUC = 0.761) and the radiomic signature (AUC = 0.919), with high sensitivity (0.957) and NPV (0.990). Among baseline characteristics, pain, facial paralysis, and reduced LMR were significantly associated with malignancy. The clinical–laboratory signature maintained stable discriminative performance (AUC > 0.7) and good calibration, consistent with our prior work (22); however, its moderate discriminatory capacity underscored the need for complementary quantitative imaging features in diagnostically challenging cases.

Beyond statistical performance, the clinical role of the proposed model requires explicit definition. The model is intended as an investigational adjunctive risk-stratification instrument that supplements, rather than replaces, the existing diagnostic workup, including clinical examination, ultrasonography, CT/MRI, FNAB, multidisciplinary evaluation, and histopathologic confirmation. We propose a specific decision node for its integration: after contrast-enhanced CT imaging and preoperative blood tests (including LMR) have been completed, and before the decision to perform FNAB or proceed directly to surgery. At this juncture, the clinician possesses CT images, blood biomarkers, and clinical findings but has not yet determined whether an invasive biopsy is warranted. In current practice, this decision rests on the clinician’s subjective synthesis of imaging findings and individual experience; the model contributes an objective, quantitative risk estimate that augments this judgment.

The model is designed to inform two distinct clinical actions, each with an appropriate evidentiary threshold. When the model yields a low-risk prediction, the high NPV (0.990) indicates that malignancy is sufficiently unlikely that FNAB may reasonably be deferred, and the patient may proceed to planned surgery without an invasive biopsy. Conversely, a high-risk prediction should prompt FNAB for cytopathologic confirmation: given the model’s modest PPV (0.449 at the cohort prevalence of 16.5%), a high-risk score alone does not justify altering surgical management without confirmatory testing. These two outputs correspond to rule-out and rule-in functions, respectively, and neither is intended to operate independently of clinical oversight.

Critically, the model is not designed to determine whether MRI should be performed. MRI remains the preferred modality for assessing deep lobe involvement, perineural spread, and the tumor–facial nerve relationship; the model does not provide information that would substitute for these assessments. The model operates exclusively within the CT-based diagnostic stream, and the decision to obtain MRI should continue to be guided by clinical presentation, sonographic findings, and institutional protocols.

In operational terms, the proposed modification to the current workflow can be summarized as follows. Currently, the pathway proceeds from clinical examination to CT, followed by subjective imaging assessment and the clinician’s discretionary decision regarding FNAB. In the proposed workflow, CT and blood tests are followed by the model generating an objective risk score, which the clinician integrates with imaging and clinical findings to reach an FNAB decision. The essential difference is the insertion of a quantitative, reproducible risk estimate at a critical decision node.

This proposed positioning remains a hypothesis derived from retrospective data. The present study was not designed to determine whether the model can support surgical decision-making without FNAB, MRI, or other established diagnostic components, and the available data are insufficient to support such a conclusion. The model’s modest PPV at low disease prevalence further reinforces that high-risk predictions require cytopathologic confirmation. Whether model-guided triage can safely reduce unnecessary FNAB procedures without missing malignancies, and whether it provides incremental value beyond expert radiologic assessment alone, are questions that require prospective evaluation.

### 4.2 CT radiomics in the context of current parotid imaging

Venous-phase CT offers uniform contrast distribution and facilitates standardized radiomics analysis (33), with studies confirming stronger performance over non-contrast CT for parotid tumor discrimination (34). In this study, 13 radiomic features were identified, and all 11 machine learning models achieved AUCs above 0.7, with XGBoost, LightGBM, and GradientBoosting above 0.9 in cross-validation. However, PPV was consistently low across individual radiomic models (range: 0.333–0.600), with false-positive rates of 40%–66.7% in the internal validation set, indicating that the modest PPV is inherent to the radiomic approach rather than specific to any single model.

As noted above, the present CT-based model is not intended to diminish the established role of MRI. Rather, it is positioned for clinical scenarios where CT is the primary or sole cross-sectional modality available, as is common in many settings where cost or access constrains MRI utilization. The findings should not be interpreted as suggesting that CT radiomics can substitute for MRI, FNAB, or histopathologic diagnosis. Future work should evaluate whether MRI-based radiomics, alone or combined with the markers assessed here, can further improve accuracy.

### 4.3 Radiomic feature interpretation

The radiomic model incorporated morphological, textural, and first-order features. The original shape sphericity feature (original_shape_Sphericity) served as the highest-weighted CT morphological feature (weight = −0.125) and may reflect tumor geometric roundness independently of gray-level information. Lower sphericity values, indicating more irregular tumor morphology, were associated with higher predicted malignancy risk. This shape feature is relatively insensitive to scanner variability (8, 34). Representative examples illustrating the segmented tumor regions are shown in **Supplementary Figures 3** and **4** for a benign parotid mass (Warthin tumor) and a malignant parotid mass (mucoepidermoid carcinoma), respectively. Each figure displays the original contrast-enhanced venous-phase CT image (Panel A), the CT image with the manually delineated VOI overlay (Panel B), and the three-dimensional tumor VOI reconstruction (Panel C). Qualitatively, the malignant lesion exhibited more irregular morphology and lower sphericity compared with the benign lesion, consistent with the quantitative radiomic findings. Additional texture features derived from GLSZM, GLCM, LBP, and wavelet transforms helped capture intratumoral heterogeneity beyond visual assessment (35).

We note that several radiomic features identified in this study have been reported to show associations with immune-related molecules in other tumor types and separate cohorts. For instance, shape-based sphericity has been associated with PD-L1 expression in non-small cell lung cancer (36) and with CD44 expression in high-grade gliomas (37); GLSZM-based textural features have shown predictive value for CD44 in gliomas (38, 39); and first-order kurtosis features have been linked to IL1B expression in head and neck squamous cell carcinoma (40). However, these associations were established in different tumor types and patient populations and were not directly investigated in the present study. No immunohistochemical validation, tumor-infiltrating lymphocyte analysis, PD-L1 assessment, or cytokine profiling was performed in our cohort. Therefore, any suggestion that the current radiomic features reflect the parotid tumor immune microenvironment remains hypothesis-generating and requires prospective validation with paired tissue-based immune profiling.

### 4.4 Class imbalance and tumor heterogeneity

The cohort exhibited substantial class imbalance (409 benign vs. 81 malignant; ~5:1) and considerable histopathologic heterogeneity within the malignant group, including mucoepidermoid carcinoma (n = 22), adenoid cystic carcinoma (n = 9), acinic cell carcinoma (n = 8), and rarer subtypes (**Supplementary Table 2**). Despite SMOTE-based balancing and strict within-fold feature selection, models trained under such conditions may learn imaging patterns specific to dominant malignant subtypes rather than generalizable malignancy-associated features, and rare subtypes are inadequately represented. Furthermore, binary benign-versus-malignant classification may oversimplify the biologic spectrum of salivary gland tumors: certain benign entities and low-grade malignancies exhibit overlapping imaging characteristics that challenge any dichotomous scheme. Subtype-stratified analyses were precluded by limited case numbers. Future multi-institutional studies with larger cohorts are needed to enable subtype-level model development and multi-class classification.

### 4.5 LMR as a systemic inflammatory marker

The present findings confirm that reduced LMR is associated with malignant parotid masses, consistent with our prior work (22). However, as a systemic composite parameter reflecting the peripheral lymphocyte-to-monocyte balance, LMR does not directly measure tumor-specific immune responses or the intratumoral immune microenvironment. The observed statistical association with malignancy should not be interpreted as evidence of a specific immunologic mechanism. Consistent with the inconsistent discriminative performance of LMR across studies (18), alternative inflammatory markers including NLR and PLR have also been investigated for parotid tumor discrimination. The moderate and variable performance of any single hematological marker reinforces the rationale for multimodal integration: LMR contributes independent information but should be combined with other data modalities rather than used in isolation.

### 4.6 Multimodal fusion: performance, interpretation, and exploratory comparison with FNAB

Multimodal data fusion has demonstrated value across various disease domains, including Alzheimer’s disease, gynecological tumors, non-small cell lung cancer, and breast cancer (41–46); its application in parotid tumor diagnosis remains limited, primarily focusing on intra-imaging modality integration (1, 34, 47–49). While Li et al. (35) proposed a model combining clinical symptoms, ultrasound features, and radiomics, it relied on subjective interpretation, introducing inter-observer variability. Committeri et al. (18) combined inflammatory biomarkers such as NLR, PLR, SII, and SIRI with MRI-based radiomic features; however, the study was limited by a small number of participants and the absence of independent validation.

The present study integrates clinical parameters, LMR, and CT radiomic features for parotid tumor risk stratification in a relatively large cohort with internal validation. The fusion model achieved an AUC of 0.931, improving specificity (0.782) and PPV (0.449) over the radiomic signature (0.734 and 0.400, respectively), while preserving sensitivity (0.957) and NPV (0.990). Unlike the poorly calibrated radiomic model, the fusion model passed the Hosmer–Lemeshow test, and DCA confirmed greater net benefit, particularly in the intermediate-risk range. Decision-level fusion using logistic regression is advantageous in moderate-sample settings, as it avoids direct integration of heterogeneous high-dimensional features, thereby reducing model complexity and the risk of overfitting (50); the use of classic machine learning rather than deep learning ensures lower data demands and greater interpretability (51).

The model’s high sensitivity (0.957) and NPV (0.990) support its potential role as an adjunctive rule-out aid: a low-risk prediction reliably identifies patients unlikely to harbor malignancy. Conversely, the modest PPV (0.449) in the full validation cohort, reflecting the low disease prevalence (16.5%), means that fewer than half of high-risk predictions correspond to true malignancies. Even a test with high sensitivity and specificity produces a substantial absolute number of false positives when disease prevalence is low; a considerable proportion of benign lesions may therefore be incorrectly flagged as high risk, potentially leading to unnecessary patient anxiety, additional invasive diagnostic procedures, or overly extensive surgery if model predictions were acted upon without confirmatory testing. These findings reinforce that the model should not be used as a standalone diagnostic instrument and that positive predictions must be confirmed through standard pathways.

We then conducted an exploratory secondary comparison of the model against FNAB-based cytopathology in the 135 patients (27.6% of the cohort) with available preoperative FNAB data. This analysis was not designed as a head-to-head comparison, and several methodological caveats must be considered when interpreting the results. First, the FNAB subset had substantially higher malignancy prevalence than the overall cohort (45.2% vs. 16.5%), which inflates the model’s apparent PPV and precludes generalization to unselected populations. Second, the temporal sequence of FNAB relative to CT imaging could not be established for all patients, and biopsy-induced tissue changes may have altered radiomic features in some cases. Third, MSRSGC categories were assigned retrospectively from non-standardized clinical reports; the concordance between retrospective and prospective Milan categorization has not been systematically evaluated, and interobserver variability in salivary gland cytopathology is well documented (52). Fourth, this comparison evaluates the model against standalone FNAB, whereas in clinical practice cytopathology is interpreted alongside imaging and physical examination within a multidisciplinary framework, which likely achieves higher integrated accuracy. Fifth, the model was applied without re-training or threshold adjustment, meaning the cutoff was not optimized for this subpopulation. Sixth, core needle biopsy offers higher sensitivity and lower nondiagnostic rates than FNAB (3), and the present analysis does not evaluate whether the model adds value beyond CNB-based diagnosis. Given these caveats, the available data are insufficient to support conclusions regarding diagnostic equivalence, superiority, or the replacement of FNAB by the proposed model; this comparison is presented solely as hypothesis-generating.

With these limitations acknowledged, the model demonstrated higher statistical discrimination than retrospectively assigned MSRSGC categories in this subset (AUC 0.952 vs. 0.712; p < 0.001). MSRSGC sensitivity in this cohort (0.410) was lower than the pooled estimate of approximately 0.78 reported in meta-analyses (3). Three factors likely account for this gap: the stringent binary threshold—only “suspicious for malignancy” and “malignant” were considered test-positive; this threshold prioritizes specificity; the enrichment of low-grade histologies whose bland cytology mimics benign aspiratess (4); and operator-dependent sampling variability. MSRSGC’s high specificity (0.986) confirms its established value as a rule-in test; its low sensitivity demonstrates that a negative FNAB does not exclude malignancy.

Model-based risk estimates differed from MSRSGC categorization in clinically meaningful directions. Among malignant cases, 79% (27/34) initially classified as low-risk by MSRSGC were classified as high-risk by the model; FNAB alone would not have raised suspicion of malignancy in these patients, and delayed diagnosis of salivary gland carcinoma can lead to inadequate resection margins and more extensive revision surgery. Conversely, among benign cases, all four cases classified as high-risk by MSRSGC were classified as low-risk by the model, though the small number of cases precludes definitive conclusions. The categorical NRI (0.399, 95% CI: 0.243–0.556) and IDI (0.386, 95% CI: 0.289–0.482) quantify this bidirectional reclassification. However, these indices should be interpreted as measures of statistical reclassification within a retrospectively defined subset, not as evidence of clinical superiority of the model over FNAB.

The model and MSRSGC exhibit complementary rather than competing performance profiles: the model provides high sensitivity (0.967) and NPV (0.968), whereas MSRSGC provides high specificity (0.986) and PPV (0.962). This complementarity motivates a hypothetical sequential risk-assessment framework—requiring prospective evaluation—in which model-based screening precedes cytopathologic confirmation. Patients classified as low-risk by the model (NPV = 0.968) might reasonably proceed without additional invasive testing, while those classified as high-risk would undergo confirmatory FNAB, where a positive result carries high diagnostic confidence. Among the 10 patients with indeterminate FNAB results (five non-diagnostic, five atypia of undetermined significance), the model’s risk estimates were concordant with final histopathology in seven cases, suggesting a potential role when cytology is equivocal, although the small sample precludes formal statistical analysis. This hypothetical sequential framework requires prospective validation.

### 4.7 Limitations and future directions

This study has several limitations, most of which reflect its retrospective, single-center design. The cohort was drawn from a tertiary referral center and comprised exclusively surgically resected cases, which may not represent the full clinical spectrum of parotid masses and may overestimate model performance. Clinical data were collected by multiple clinicians over a 10-year period without prospective standardization, and the temporal relationship among FNAB, blood sampling, and CT imaging was inconsistently documented. Acquisition-related variability across CT scanners and protocols over the same period could not be fully eliminated, and ComBat harmonization was not applied. Manual tumor segmentation, despite acceptable reproducibility (ICC > 0.75), introduces subjectivity. The FNAB comparison was limited to 135 patients (27.6%) with enriched malignancy prevalence, and MSRSGC categories were assigned retrospectively from non-standardized reports. The model additionally lacks paired tissue-based immune characterization, precluding mechanistic interpretation of imaging–immune relationships, and class imbalance (5:1) with histopathologic heterogeneity constrains subtype-level inference.

These limitations are largely inherent to the retrospective design and cannot be adequately corrected through *post hoc* statistical adjustment. They are best addressed at the prospective study-design stage, which should incorporate, at a minimum: predefined and consistently applied inclusion and exclusion criteria; standardized CT acquisition protocols with ComBat or equivalent harmonization across scanner platforms; standardized timing of blood collection relative to imaging; systematic documentation of all procedural interrelationships; consecutive patient enrollment to minimize selection bias; *a priori* statistical analysis plans with prespecified endpoints; and at least one independent external validation cohort.

More broadly, improved statistical discrimination—as reflected by higher AUC, sensitivity, or specificity—does not automatically establish clinical utility. The translation from retrospective model performance to measurable improvements in patient management, diagnostic efficiency, or clinical outcomes requires progressive validation stages: independent external validation across different institutions and patient populations; prospective evaluation with predefined protocols and consecutively enrolled patients; demonstration of incremental value beyond the standard diagnostic workup (including clinical examination, CT/MRI interpretation, FNAB/CNB, and multidisciplinary assessment); assessment of clinical utility, including measurable impact on patient-relevant outcomes; and ultimately incorporation into evidence-based clinical practice guidelines. The present study addresses only the initial derivation and internal validation stage. None of the subsequent stages has been addressed, and the current findings should therefore be interpreted as preliminary and hypothesis-generating rather than practice-changing. Definitive conclusions regarding the clinical value of this approach await prospectively designed multicenter studies.

## 5 Conclusions

In conclusion, a multimodal model integrating clinical features, LMR, and CT radiomic features was developed and internally validated for preoperative risk stratification of parotid masses, demonstrating improved statistical discrimination over single-modal approaches, with high sensitivity and NPV and modest PPV. The model represents an investigational adjunctive risk-stratification instrument intended to supplement, rather than replace, established diagnostic modalities, including FNAB, MRI, and histopathologic assessment. These findings are preliminary and do not establish clinical utility; prospective multicenter validation with standardized protocols and external cohorts is required before clinical consideration.

## Data Availability

The raw data supporting the conclusions of this article will be made available by the authors, without undue reservation.

## Supplementary material

Supplementary Figure 1 Segmentation of three-dimensional volume of interest (VOI).

Supplementary Figure 2 Types and statistics of radiomics features. **(A)** Radiomics features and proportion of various types; **(B)** comparison of radiomics features between benign and malignant masses.

Supplementary Figure 3 Representative benign parotid mass (Warthin tumor). **(A)** Original venous-phase contrast-enhanced CT image; **(B)** CT image with manual volume-of-interest (VOI) segmentation overlay; **(C)** Three-dimensional VOI reconstruction.

Supplementary Figure 4 Representative malignant parotid mass (mucoepidermoid carcinoma). **(A)** Original venous-phase contrast-enhanced CT image; **(B)** CT image with manual VOI segmentation overlay; **(C)** Three-dimensional VOI reconstruction.
