# Supplementary figures and images for: Could a multimodal fusion model integrating CT radiomics and systemic inflammatory markers improve preoperative risk stratification of parotid masses? A retrospective exploratory study
Source: Front Immunol. 2026 Jul 1;17:1842957. doi: 10.3389/fimmu.2026.1842957 (PMC13369007; doi:10.3389/fimmu.2026.1842957)

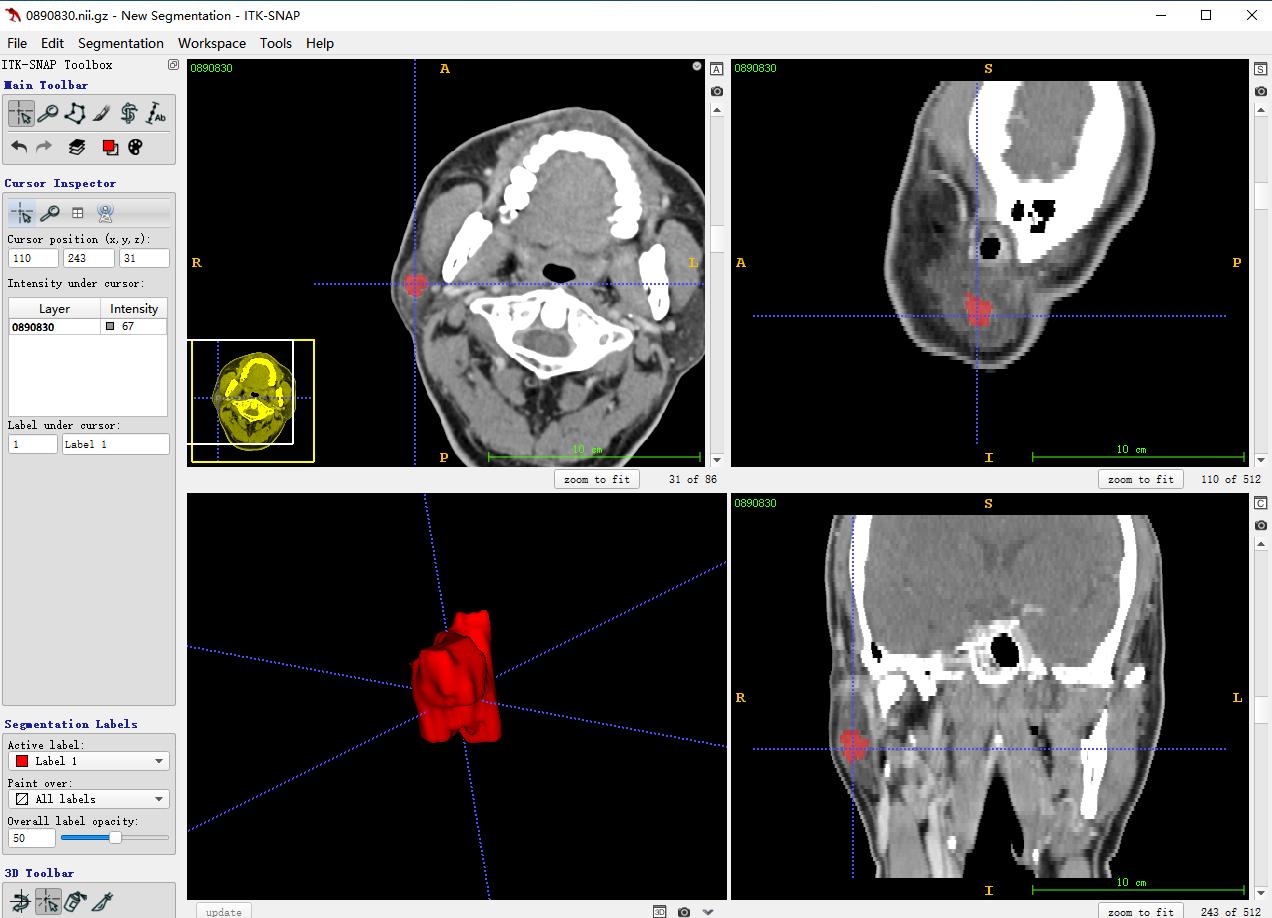

Supplement: Supplementary Figure 1 — Segmentation of three-dimensional volume of interest (VOI). [file Image1.jpeg]

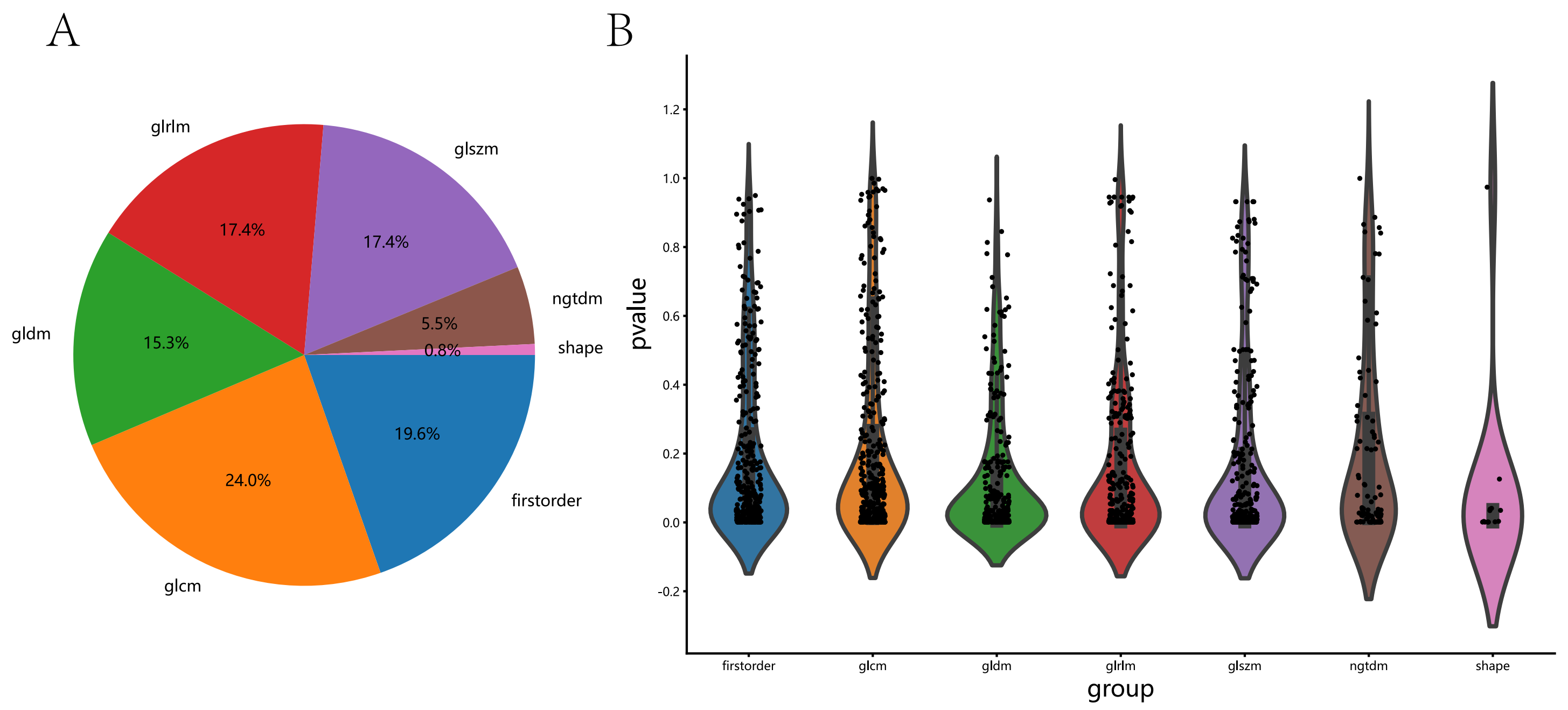

Supplement: Supplementary Figure 2 — Types and statistics of radiomics features. (A) Radiomics features and proportion of various types; (B) comparison of radiomics features between benign and malignant masses. [file Image2.tif]

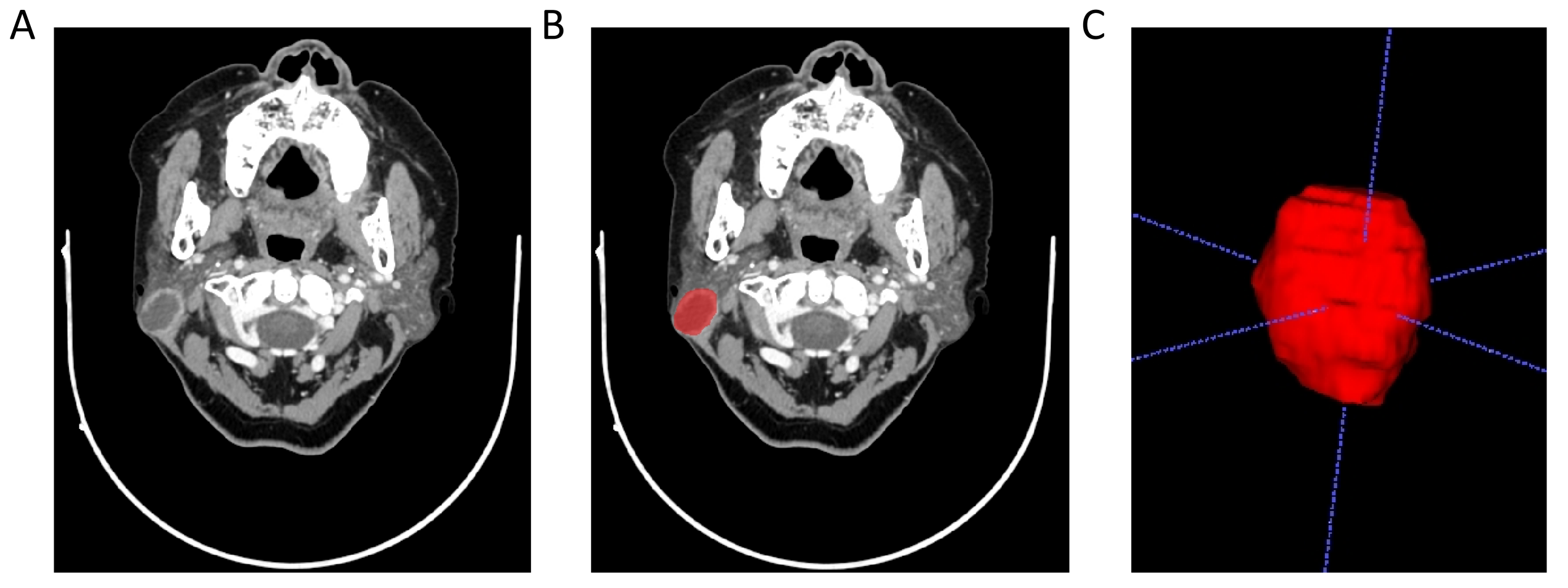

Supplement: Supplementary Figure 3 — Representative benign parotid mass (Warthin tumor). (A) Original venous-phase contrast-enhanced CT image; (B) CT image with manual volume-of-interest (VOI) segmentation overlay; (C) Three-dimensional VOI reconstruction. [file Image3.tiff]

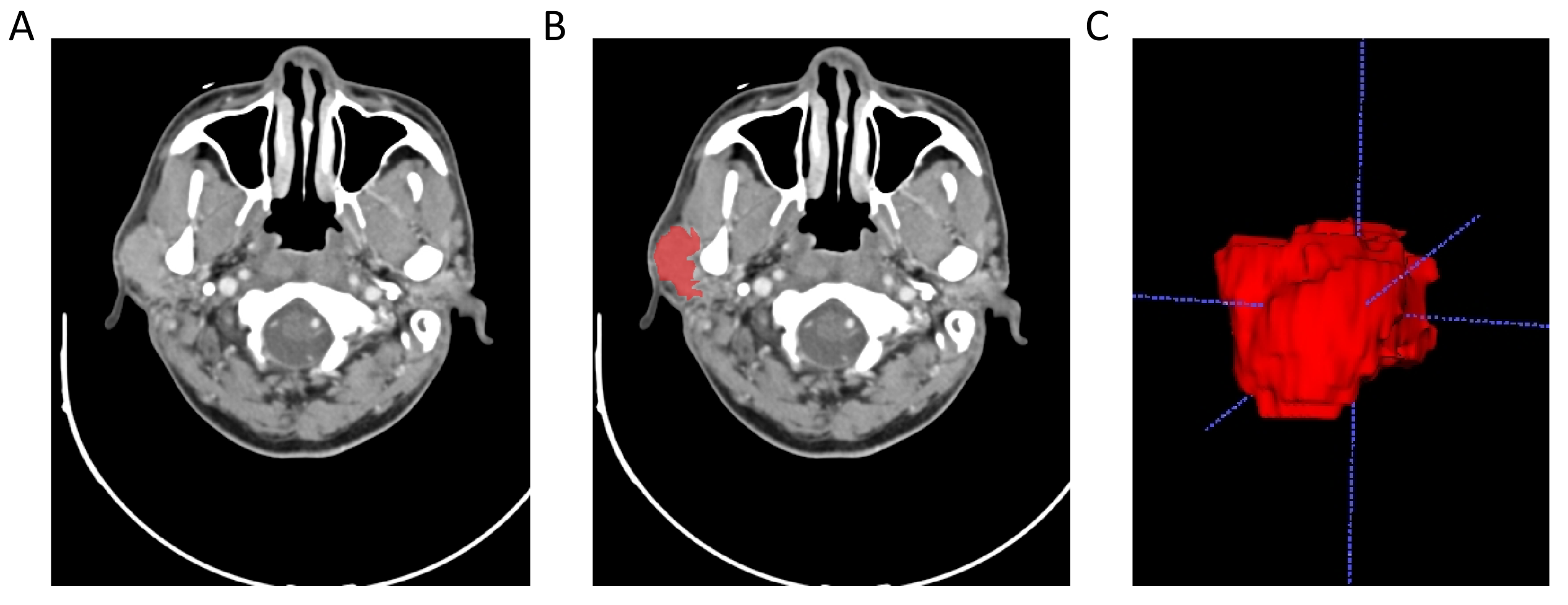

Supplement: Supplementary Figure 4 — Representative malignant parotid mass (mucoepidermoid carcinoma). (A) Original venous-phase contrast-enhanced CT image; (B) CT image with manual VOI segmentation overlay; (C) Three-dimensional VOI reconstruction. [file Image4.tiff]
